# Mechanisms of antimicrobial resistance: From genetic evolution to clinical manifestations

**DOI:** 10.3934/microbiol.2025045

**Published:** 2025-12-18

**Authors:** Sabiha Nusrat, Mansur Aliyu, Fatema Tuz Zohora

**Affiliations:** 1 Sabiha Nusrat, Department of Biological Sciences, Texas Tech University, Lubbock, Texas, USA; 2 Mansur Aliyu, Department of Medical Microbiology and Parasitology, Faculty of Basic Clinical Science, College of Health Sciences, Bayero University, Kano, Nigeria; 3 Fatema Tuz Zohora, Jeffrey Cheah School of Medicine and Health Sciences, Monash University Malaysia,47500, Subang Jaya, Selangor, Malaysia; The Quest, Chittagong, Bangladesh

**Keywords:** antimicrobial resistance, multidrug resistance, gene transfer, biofilms, porins

## Abstract

Antimicrobial resistance (AMR) is a significant global health challenge that threatens the effectiveness of antibiotics and other antimicrobial agents. Here, we examined the molecular mechanisms that contribute to bacterial resistance, including alterations at target sites, enzymatic inactivation, efflux pump overexpression, and biofilm formation. Key resistance determinants, such as *bla*_CTX-M-15_, *bla*_NDM-1_, *mecA*, and *erm* genes, mediate enzymatic degradation and target modification, thereby diminishing antibiotic potency. Clinically significant pathogens, including *Escherichia coli*, *Pseudomonas aeruginosa*, *Klebsiella pneumoniae*, *Staphylococcus aureus*, and *Enterococcus faecium*, exemplify a broad spectrum of resistance and frequently acquire these traits through horizontal gene transfer (HGT), facilitated by plasmids, integrons, and transposons. The propensity for biofilm formation further augments bacterial persistence by impeding antimicrobial penetration and fostering intra-community genetic exchanges. The clinical ramifications of AMR are profound, contributing to elevated morbidity and mortality, extended hospitalization, and increased rates of therapeutic failure, all of which exert significant strain on the healthcare system. The economic consequences are equally severe, with escalating healthcare expenditures and substantial projected losses to the global gross domestic product (GDP). Addressing these challenges necessitates the adoption of advanced approaches, including genomic surveillance, antimicrobial stewardship, novel inhibitors targeting resistance pathways, immuno-antibiotics, and bacteriophage therapy. This review underscores the need to integrate molecular diagnostics and a One Health perspective to monitor and contain resistance across human, animal, and environmental reservoirs. A comprehensive understanding of the molecular and epidemiological aspects of AMR is essential for driving advancements in diagnostics, therapeutics, and policies, thereby ensuring global health protection.

## 1. Introduction

Antimicrobial resistance (AMR) has rapidly become one of the most critical public health challenges of the 21st century, undermining the effectiveness of antibiotics, antivirals, antifungals, and antiparasitics, which are the cornerstones of modern medicine [1]–[3]. The World Health Organization (WHO) estimates that bacterial AMR was directly responsible for 1.27 million deaths in 2019 and contributed to nearly 5 million deaths globally, with the burden rising sharply across all regions and income levels [1],[4].

Recent analyses indicate that between 4 and 7.1 million deaths worldwide in 2021 were associated with bacterial AMR, and projections suggest that without robust interventions, annual deaths could double by 2050, potentially reaching 10 million annually [5],[6]. The economic ramifications are equally profound, with the World Bank projecting up to US$3.4 trillion in annual gross domestic product (GDP) losses by 2030 if AMR remains unchecked [1].

The misuse and overuse of antimicrobials in human medicine, agriculture, and animal husbandry fuel AMR. This problem is further exacerbated by inadequate infection prevention and control, poor sanitation, and limited access to quality diagnostics and therapeutics [1],[7],[8]. The coronavirus disease 2019 (COVID-19) pandemic has further aggravated this crisis, driving increased and often inappropriate antibiotic use, which has accelerated the emergence and spread of resistant pathogens [2],[7]. The consequences are dire: Routine infections are becoming more difficult to treat, medical procedures such as surgery and chemotherapy are riskier, and vulnerable populations, particularly children, the elderly, and immunocompromised individuals, face heightened risks of morbidity and mortality [8].

### 1.1. Epidemiological trends and the impact on public health

Epidemiological surveillance from 2020 to 2025 has revealed an alarming increase in resistance among Gram-positive and Gram-negative bacteria [2],[3]. The WHO Global AMR and Use Surveillance System (GLASS) reported a median resistance rate of 42% for third-generation cephalosporin-resistant *Escherichia coli* and 35% for methicillin-resistant *Staphylococcus aureus* (MRSA) in 76 countries [2]. Resistance to last-resort antibiotics, such as carbapenems and colistin, is increasing in pathogens, such as *Klebsiella pneumoniae* and *Acinetobacter baumannii*, rendering even the most potent drugs ineffective [9].

The clinical and economic impacts are substantial: AMR leads to prolonged hospital stays, increased healthcare costs, and higher rates of treatment failure and mortality [2],[10]. The pandemic-driven surge in antimicrobial use and global spread of resistant clones highlights the urgent need for coordinated surveillance, stewardship, and research [7],[11]. Only 1.3% of microbiology laboratories in key African states can test for priority AMR pathogens, highlighting this gap [7].

Despite growing awareness, the mechanisms by which microorganisms acquire and propagate resistance remain complex and multifaceted, involving genetic mutations, horizontal gene transfer (HGT), efflux pumps, and biofilm formation [12],[13]. Understanding these molecular and evolutionary processes is essential for developing innovative diagnostic, therapeutic, and stewardship strategies [14],[15].

In this review, we summarize recent advances (2020–2025) in the genetic and biochemical mechanisms underlying AMR, linking these insights to the clinical manifestations and epidemiological trends. By integrating data from global surveillance, molecular microbiology, and clinical practice, we aim to elucidate the pathways driving resistance and inform evidence-based interventions to mitigate this escalating crisis [16]–[18].

## 2. Genetic evolution of AMR

### 2.1. Origins and spread of resistance genes

**Figure 1.**
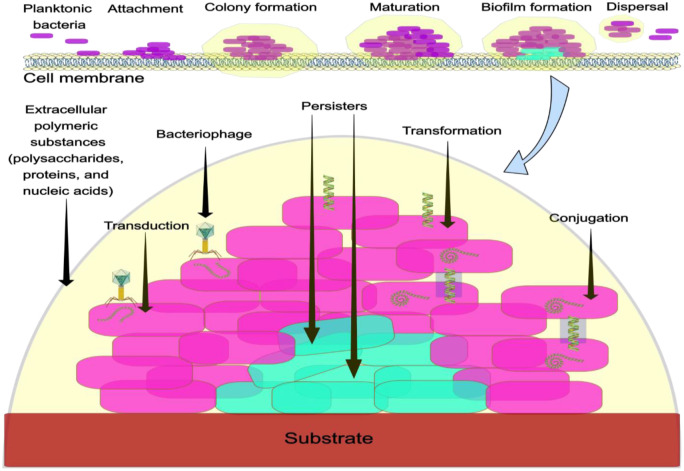
Genetic Evolution of AMR in bacteria: Mechanisms and influencing factors. This diagram provides a detailed depiction of the complex genetic processes that enable the emergence and spread of AMR in bacteria. As shown within the yellow dome, the HGT mechanisms of conjugation, transformation, and transduction among purple bacilli enable the swift acquisition of resistance determinants across various bacterial populations, which can occur among different species (not shown). The role of mobile genetic elements (MGEs), such as plasmids, transposons, and integrons, which act as vehicles for capturing, mobilizing, and integrating resistance genes into bacterial genomes, is not shown in this figure. These elements facilitate the assembly of multidrug resistance loci and promote their dissemination within and between bacterial communities. Furthermore, genetic background significantly influences the mutational pathways leading to resistance. Pre-existing genomic variations influence the fitness effects of new mutations through epistatic interactions, thereby constraining or facilitating specific evolutionary pathways. For example, in *E. coli*, mutations in genes such as *gyrA* and *parC* confer fluoroquinolone resistance; however, their phenotypic expression and evolutionary stability depend on the genetic context of the strains. This interplay between HGT, MGE integration, and genetic background underscores the dynamic and multifactorial nature of resistance evolution, informing surveillance strategies and guiding the development of targeted interventions to curb the spread of AMR. The yellow dome represents an extracellular polymeric substance composed of proteins, polysaccharides, and nucleic acids that provides a cover to limit antibiotic penetration. At the bottom of the figure are the persister cells (green rods), which are non-mutant, non-proliferative phenotypic variants that exhibit tolerance to antibiotics and are highly prevalent in biofilms. Their ability to remain dormant during stressful periods, particularly when exposed to antibiotics, makes them a significant factor in the persistence of chronic biofilm-associated infections.

The rise in AMR is driven by the genetic adaptability of bacteria, which enables them to acquire, accumulate, and disseminate resistance determinants across ecological and clinical settings. The genetic evolution of AMR is orchestrated through a complex interplay of HGT mechanisms, mobilization of resistance genes via mobile genetic elements (MGEs), and persistence of these determinants within environmental and clinical reservoirs [19]–[22], as depicted in Figure 1.

#### 2.1.1. HGT: Conjugation, transformation, and transduction

Bacteria acquire new resistance traits primarily through HGT mechanisms that enable rapid adaptation to antimicrobial pressure. Conjugation, the direct transfer of plasmid DNA between bacteria via cell-to-cell contact, is the most efficient and prevalent mode of HGT for resistance genes, particularly those encoding β-lactamases such as *bla*_CTX-M_, *bla*_NDM_, and *bla*_KPC_, as well as aminoglycoside-modifying enzymes, including aac(6′)-Ib and carbapenemases [21]–[23]. Transformation, the uptake of free DNA from the environment, further facilitates the acquisition of resistance determinants, especially in naturally competent species, such as *Streptococcus pneumoniae* and *A. baumannii*. Transduction mediated by bacteriophages enables the transfer of resistance genes embedded within phage genomes, contributing to the spread of AMR in clinical and environmental reservoirs [24].

#### 2.1.2. MGEs: Plasmids, transposons, and integrons

The mobility of resistance genes is underpinned by MGEs, including plasmids, transposons, and integrons, which serve as vehicles for gene capture and dissemination of resistance genes. Plasmids, which are small, circular, and double-stranded self-replicating DNA molecules, frequently harbor clusters of resistance genes and facilitate their horizontal spread across bacterial species and genera [21],[22]. Transposons, or “jumping genes,” can excise and integrate into different genomic or plasmid locations, often carrying multiple resistance determinants, such as *tet(M)* for tetracycline resistance and *vanA* for vancomycin resistance [23],[25].

Integrons are genetic platforms capable of capturing, incorporating, and expressing gene cassettes, particularly those encoding antibiotic resistance, enabling bacteria to rapidly adapt to antimicrobial pressure. Hence, they play a pivotal role in the assembly and mobilization of multidrug-resistant (MDR) loci. Class 1 integrons are widely associated with resistance to aminoglycosides, sulfonamides, and β-lactams and are frequently linked to transposons and plasmids, amplifying their impact on AMR dissemination [20],[26].

#### 2.1.3. Environmental and clinical reservoirs

The origin and persistence of resistance genes are intricately linked to environmental and clinical reservoirs. Environmental biofilms, wastewater treatment plants, agricultural runoff, and animal husbandry are rich reservoirs of resistance genes and MGEs. Studies have shown that sub-inhibitory concentrations of antibiotics in these environments select for resistant bacteria and promote HGT, especially within biofilm communities, where cell density and genetic exchange rates are high. Environmental viromes, particularly bacteriophages, are increasingly recognized as vectors for the transfer of resistance genes, further complicating the resistome landscape [24]. In clinical settings, the convergence of high antibiotic use and dense microbial populations accelerates the selection and spread of MDR clones, such as *E. coli* ST131 and *K. pneumoniae* ST258, which harbor plasmid-encoded resistance genes [21].

Genomic studies have revealed that the evolution of resistance is not solely a function of gene acquisition but also of clonal expansion, intra-clone diversification, and genetic convergence, as observed in dominant MDR lineages [25]. The interplay between phenotypic resistance and underlying genotypic traits underscores the complexity of AMR evolution. Ultimately, the dynamic exchange of resistance genes across environmental and clinical reservoirs, mediated by HGT and MGEs, sustains the global AMR crisis and necessitates integrated surveillance and mitigation strategies [17],[21]–[23].

### 2.2. Genomic evolution and historical contingency

The evolutionary trajectory of AMR is profoundly shaped by the genetic background and unique array of pre-existing mutations within bacterial strains. Genomic studies have revealed that even minor differences in genetic backgrounds can lead to divergent evolutionary outcomes under identical antibiotic selection pressures [17]. This phenomenon, termed historical contingency, demonstrates that resistance evolution is not deterministic but contingent on prior evolutionary history.

For instance, *E. coli* strains separated by fewer than 100 mutations exhibit idiosyncratic responses to antibiotics, such as ampicillin and ciprofloxacin, with some backgrounds showing a constrained evolutionary potential for resistance [27]. Crucially, the interplay between these pre-existing mutations often manifests as epistatic interactions, further modulating resistance evolution within each unique genetic background.

Epistatic interactions, in which the fitness effect of a mutation is contingent on the genetic context, play a pivotal role in shaping mutational pathways that lead to AMR. One well-characterized pattern is diminishing return epistasis, exemplified by early resistance mutations, such as those in *gyrA* conferring ciprofloxacin resistance, which provide substantial fitness advantages, whereas subsequent mutations yield progressively smaller benefits, thereby constraining long-term resistance evolution [28],[29]. Negative epistasis is observed between mutations in *gyrA* and *gyrB*, both of which encode DNA gyrase subunits (type II topoisomerase) that are often mutually exclusive because double mutants do not confer additional fitness under ciprofloxacin selection [27].

Conversely, positive epistasis can occur in MDR bacteria, where synergistic interactions between mutations, such as efflux pump overexpression combined with target site modifications, enhance resistance levels beyond the additive effects [20]. These epistatic relationships effectively create “genetic channels,” that either restrict or facilitate specific evolutionary trajectories. For instance, fluoroquinolone resistance in *S. pneumoniae* necessitates mutations in *parC* (topoisomerase IV) prior to *gyrA* (DNA gyrase), as the reverse sequence results in reduced fitness, highlighting the importance of the mutation order [17].

Similarly, carbapenem resistance in *P. aeruginosa* is shaped by epistatic interactions between *oprD* (porin loss) and *ampC* (β-lactamase overexpression), where combined alterations potentiate resistance more effectively than individual mutations [30]. Together, these findings underscore the complexity of resistance evolution governed by epistasis, which has significant implications for predicting and managing AMR.

### 2.3. Emergence of novel resistance genes

The last five years have witnessed accelerated discovery and global dissemination of novel AMR gene families, driven by advances in whole-genome sequencing (WGS) and metagenomics. Research has uncovered a wide array of resistance genes across human, animal, aquatic, and environmental reservoirs, underscoring the interconnected nature of AMR emergence and the critical need for comprehensive surveillance [31],[32].

Aminoglycoside resistance genes, such as *aac(3)-IId*, *aph(3″)-Ib*, *aph(6)-Id*, *aadA1*, and *aadA5*, are highly prevalent in isolates from humans, livestock, fish, and environmental samples, reflecting the widespread HGT and selection pressure in clinical and agricultural settings. Similarly, the β-lactamase gene *bla*_CTX-M-15_, which confers resistance to third-generation cephalosporins, has become pervasive globally and has been identified in nearly all the sampled environments across continents [31].

Genomic surveillance has revealed the swift dissemination of quinolone resistance mutations, such as *gyrA*_S83L, *gyrA*_D87N, and *parC*_S80I, and tetracycline resistance genes, including *tet(A)*, *tet(B)*, and *tet(D)* across ecological niches. This highlights the role of mobile plasmids, including IncFIA, IncI1, and IncFII, in facilitating gene exchange among humans, livestock, and aquatic environments [31].

The global movement of people, animals, and goods further amplifies the dissemination of these resistance determinants, with recent evidence showing that travelers can carry resistant bacteria for months after exposure to endemic regions, contributing to their international spread [33]. Collectively, these findings illustrate the dynamic evolution and worldwide transmission of novel AMR genes, emphasizing the urgency of the One Health approach and advanced molecular diagnostics for monitoring and containing emerging threats [31]–[33].

## 3. Molecular mechanisms of AMR

**Figure 2.**
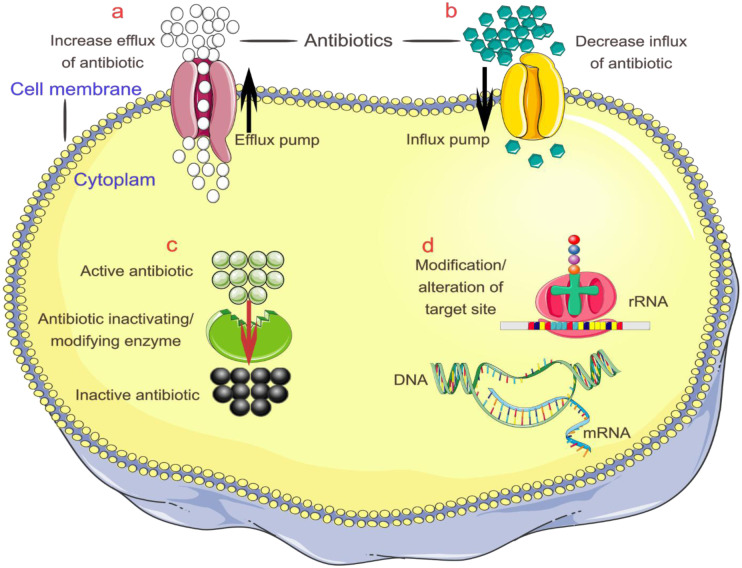
Schematic overview of the four principal mechanisms of AMR in bacteria. This figure illustrates the primary molecular strategies employed by bacteria to evade antimicrobial agents. (a) Active efflux is mediated by efflux pumps embedded in the bacterial membrane, such as those belonging to the RND, MFS, SMR, and ABC families. These porins actively transport a broad spectrum of antibiotics out of the cell, maintain sub-inhibitory intracellular levels, and contribute to MDR. (b) The limitation of drug uptake is depicted through alterations or loss of outer membrane porins (influx pumps), particularly in Gram-negative bacteria, which reduces the entry of hydrophilic antibiotics and consequently lowers intracellular drug concentrations. (c) Enzymatic inactivation involves the production of specific enzymes, such as β-lactamases, aminoglycoside-modifying enzymes, and chloramphenicol acetyltransferases, which chemically modify or degrade antibiotics, rendering them ineffective (inactive). (d) Target modifications are visualized as genetic mutations or post-translational modifications in antibiotic targets, including alterations in ribosomal RNA, penicillin-binding proteins, or DNA gyrase, which reduce drug binding and efficacy. The fifth AMR mechanism, biofilm formation, is shown in Figure 1.

The molecular mechanisms driving AMR in bacteria are intricately complex, as illustrated in Figure 2, and collectively undermine the efficacy of essential therapies. Resistance often arises through enzymatic degradation or modification of antibiotics, such as β-lactamases, alteration of antibiotic-binding targets, and active efflux systems that expel drugs from cells [11].

Furthermore, changes in membrane permeability reduce drug uptake, and biofilm formation shields bacterial communities from antimicrobial agents, thereby promoting persistent infection [17],[24]. Genomic studies suggest that these mechanisms frequently evolve concurrently, enhancing multidrug resistance and complicating clinical management [21],[34]. Table 1 summarizes the mechanisms of bacterial AMR.

**Table 1.** Molecular mechanisms of AMR.

Mechanism	Description	Molecular Basis	Representative Examples	Reference
Uptake Limitation	Reduction or alteration of membrane permeability to prevent antibiotic entry into bacterial cells	Downregulation or mutation of porin genes such as *OmpF*, *OmpC*, *OprD*, changes in OMPs, biofilm formation	*P. aeruginosa* downregulates OprD porin causing carbapenem resistance; *E. coli* modifies OmpF channels reducing β-lactam uptake	[34]–[36]
Efflux Pumps	Active extrusion of antibiotics from the bacterial cell to maintain sub-inhibitory intracellular concentrations	Overexpression of efflux pump families: RND, MFS, ABC, SMR, MATE	*A. baumannii* employs RND pumps (AdeABC) to expel aminoglycosides and carbapenems; *K. pneumoniae* uses AcrAB-TolC efflux system for MDR	[34],[36],[37]
Enzymatic Inactivation	Bacterial enzymes chemically modify or degrade antibiotics, rendering them ineffective	Production of β-lactamases such as ESBLs, carbapenemases like KPC, NDM, aminoglycoside-modifying enzymes, chloramphenicol acetyltransferases	*K. pneumoniae* produces KPC carbapenemase hydrolyzing carbapenems; *S. aureus* expresses β-lactamase BlaZ hydrolyzing penicillins	[34],[35],[38]
Target Modification	Mutation, modification, or protection of antibiotic targets to reduce drug binding and efficacy	Point mutations in genes encoding targets (*gyrA*, *parC* for fluoroquinolones), methylation of 23S rRNA by *erm* genes, acquisition of altered PBPs (PBP2a)	*S. aureus* acquires *mecA* gene encoding PBP2a conferring methicillin resistance; *Enterococcus faecium* methylates 23S rRNA causing macrolide resistance	[34],[35],[38]
Biofilm Formation and Resistance	Bacterial communities encased in a self-produced extracellular matrix exhibit heightened resistance to antimicrobials	Biofilm matrix limits antibiotic penetration, harbors antibiotic-modifying enzymes (β-lactamases), promotes slow growth/persister cells, enhances efflux pump expression, facilitates HGT	*P. aeruginosa* biofilms show increased β-lactamase activity and efflux pump expression; *K. pneumoniae* biofilms degrade ampicillin via matrix β-lactamase; *S. aureus* biofilms display tolerance due to persister cells and altered metabolism	[39]–[43]

OMP—Outer membrane proteins; RND—Resistance nodulation division family; MFS—Major facilitator superfamily; ABC—ATP binding cassette; SMR—Small multidrug resistance; MATE—Multidrug and toxic compound extrusion; ESBLs—Extended spectrum beta lactamases; KPC—*Klebsiella pneumoniae* carbapenemase; NDM—New Delhi metallo-beta-lactamase; PBSs –Penicillin binding proteins; rRNA—Ribosomal ribonucleic acid; HGT—Horizontal gene transfer.

### 3.1. Limiting drug uptake

Gram-negative bacteria employ outer membrane modifications, particularly porin alterations, to limit antimicrobial entry, thereby evading antibiotic activity. Porins, such as OmpF and OmpC in *E. coli*, OprD in *P. aeruginosa*, and OmpK35/OmpK36 in *K. pneumoniae*, function as channels for the passive diffusion of hydrophilic antibiotics. Mutations that lead to the downregulation, loss, or structural changes in these porins significantly reduce the membrane permeability. For example, the loss or modification of OprD in *P. aeruginosa* decreases carbapenem uptake, contributing to carbapenem resistance, whereas alterations in OmpK36 in *K. pneumoniae* confer resistance to cephalosporins and carbapenems [44]–[46]. Clinically, porin-deficient strains are associated with MDR phenotypes and poor therapeutic outcomes, especially when combined with β-lactamase production, highlighting the critical role of limited drug uptake in the evolution of resistance [47],[48].

### 3.2. Active efflux of antimicrobials

Active efflux pumps constitute another major mechanism by which bacteria reduce intracellular antibiotic concentrations. These transporters are grouped into four primary families based on their structure and energy source: Resistance-nodulation-division (RND), major facilitator superfamily (MFS), small multidrug resistance (SMR), and ATP-binding cassette (ABC) transporters [49],[50]. RND pumps, such as AcrAB-TolC in *E. coli* and MexAB-OprM in *P. aeruginosa*, span the inner membrane, periplasm, and outer membrane and utilize the proton motive force to expel a broad range of antibiotics, including β-lactams, fluoroquinolones, and tetracyclines [51],[52].

The NorA pump in *S. aureus*, a type of MFS pump, primarily expels fluoroquinolones using proton gradient. In contrast, SMR transporters typically target antiseptics, and ABC transporters hydrolyze ATP to export macrolides and peptides [53],[54]. Overexpression of efflux pumps, often regulated by transcriptional activators such as MarA and SoxS, significantly contributes to MDR by synergizing with other resistance mechanisms, thus complicating treatment strategies [55],[56].

### 3.3. Enzymatic inactivation of drugs

Enzymatic inactivation remains one of the most sophisticated bacterial defense strategies against antibiotic treatment. β-Lactamases are the most extensively studied enzymes, classified into four Ambler classes: Class A (CTX-M-15 extended-spectrum β-lactamases [ESBLs]), Class B metallo-β-lactamases (NDM, VIM), Class C (AmpC cephalosporinases), and Class D (OXA-type carbapenemases). These enzymes hydrolyze β-lactam antibiotics, rendering them ineffective [57]–[59].

Aminoglycoside resistance is primarily mediated by aminoglycoside-modifying enzymes (AMEs), including acetyltransferases (AAC), phosphotransferases (APH), and nucleotidyltransferases (ANT), which chemically modify aminoglycosides and prevent their binding to ribosomal targets [60],[61]. Additionally, other enzymes, such as chloramphenicol acetyltransferases and macrolide phosphotransferases, contribute to resistance against their respective antibiotic classes [34],[62],[63].

The evolution and diversification of these enzymes are driven by gene mutations, duplication events, and HGT, which are facilitated by plasmids, integrons, and transposons. The global dissemination of genes such as *bla*_CTX-M-15_ and *bla*_NDM-1_ exemplifies the rapid spread of enzymatic resistance determinants, often in combination with porin loss or efflux pump overexpression, resulting in highly resistant clinical isolates with limited treatment options [64],[65]. This multifaceted enzymatic arsenal underscores the complexity of AMR and highlights the urgent need for novel therapeutic and diagnostic strategies to combat AMR.

### 3.4. Modification of drug targets

Bacteria evade antimicrobials through structural alterations in their target drugs. Ribosomal modifications, particularly in 16S rRNA and ribosomal proteins, compromise the binding of aminoglycosides and tetracyclines. Mutations in DNA gyrase genes, such as *gyrA*, reduce fluoroquinolone efficacy by diminishing drug–enzyme affinity [66],[67]. Penicillin-binding protein (PBP) mutations or the acquisition of low-affinity PBPs, such as PBP2a, in MRSA confer β-lactam resistance by disrupting the binding of antibiotics. Beyond classical mechanisms, CRISPR-Cas systems contribute to resistance via adaptive immunity against MGEs, although their role remains secondary to plasmid-mediated HGT [68]–[70].

### 3.5. Biofilm formation and resistance

Biofilms are structures formed by microorganisms that attach to surfaces through self-produced extracellular matrices. This matrix contains extracellular polymeric substances (EPS), including polysaccharides, proteins, and nucleic acids, which protect bacteria from threats [71],[72]. Biofilms enhance bacterial survival and cause chronic infections, resulting in increased resistance through restricted antibiotic penetration and formation of persister cells [73],[74]. Biofilms enhance tolerance through multiple mechanisms. EPS limits antibiotic penetration (Figure 1), whereas metabolic heterogeneity creates resistant and dormant subpopulations.

Chronic infections are caused by the biofilms of *P. aeruginosa* and *S. aureus*, which utilize multiple mechanisms to achieve antibiotic resistance [75]–[77]. Chronic lung infections in patients with cystic fibrosis feature *P. aeruginosa* biofilms that use efflux pumps and stress-response pathways. Similarly, *S. aureus* forms biofilms on medical devices, such as catheters and prosthetic joints. Biofilms generally evade immune clearance and resist therapy by limiting antibiotic penetration and harboring persister cells. This tolerance necessitates the use of adjunct therapies that disrupt biofilms to treat chronic infections, such as cystic fibrosis and wounds [78],[79].

**Figure 3.**
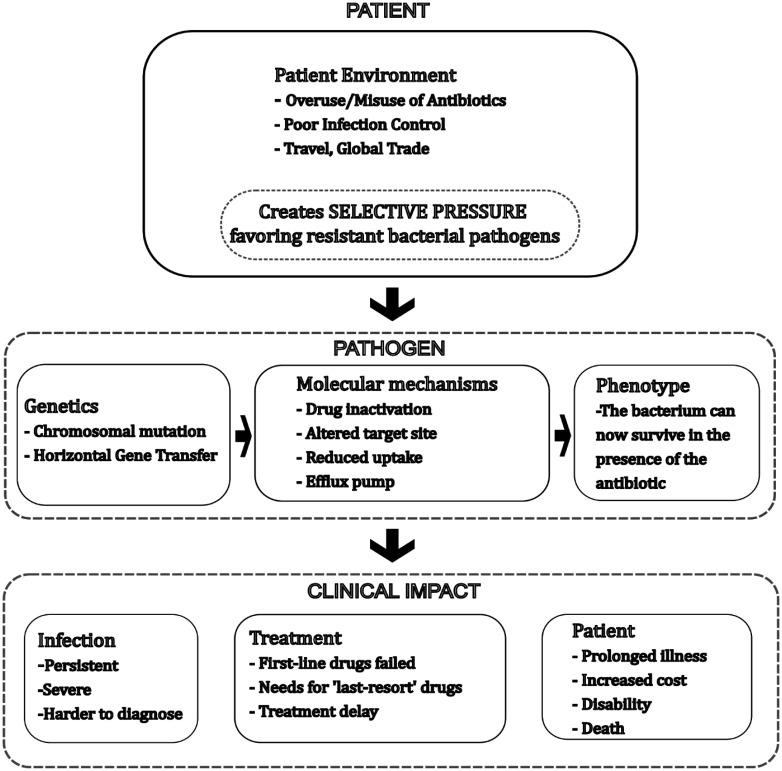
Schematic overview of the development and clinical impact of antibiotic resistance in bacterial pathogens. This schematic diagram illustrates the sequence of events and mechanisms leading from patient-related factors to the clinical consequences of antibiotic-resistant bacterial infection. It begins with patient environmental factors, such as antibiotic overuse, poor infection control, and global travel, which generate selective pressure favoring resistant pathogens. In the pathogen section, genetic mechanisms such as chromosomal mutation and horizontal gene transfer drive molecular mechanisms (e.g., drug inactivation, altered drug targets, reduced uptake, and efflux pumps) that enable bacteria to survive antibiotic exposure. The clinical impact is depicted across three domains: Persistent infection that is difficult to diagnose, limited or delayed treatment options due to failed first-line drugs, and prolonged illness, higher costs, disability, or death.

Biofilm formation involves multiple genes and regulatory pathways. In *P. aeruginosa*, *las* and *rhl* quorum-sensing systems regulate biofilm formation through autoinducers. The *pel* and *psl* genes produce polysaccharides with a matrix structure [80],[81]. In *S. aureus*, the *icaADBC* operon enables the synthesis of intercellular polysaccharide adhesin for biofilm formation. Mutations that enhance the expression of these genes increase biofilm persistence [82],[83]. Biofilm infections require a combination of antibiotics and device removal to be treated. Understanding the mechanisms of biofilm formation is essential for developing preventive strategies [84],[85]. Figure 3 provides a schematic view of the interplay between the genetic, molecular, and clinical aspects of AMR.

## 4. Clinical manifestations and implications

Continuous advancements in AMR have significantly altered the field of infectious diseases, converting previously manageable infections into challenging clinical conditions. A comprehensive understanding of the direct and indirect effects of resistance on patient outcomes, along with persistent diagnostic challenges, is crucial for effective infection management and safeguarding global health [86]–[88]. Table 2 shows the clinical effects of AMR on specific bacterial pathogens.

**Table 2.** Clinical impact of AMR.

Pathogen	Key Resistance Trends (2020–2025)	Clinical Outcomes & Impact	Notable Outbreaks/Data Points	Reference
*E. coli*	425 median resistance to third-generation cephalosporins. High resistance to ampicillin, co-trimoxazole, fluoroquinolones (1 in 5 UTI cases in 2020). Increasing prevalence of ESBL (CTX-M) and carbapenemase (NDM-5, OXA-181) producers.	Harder-to-treat urinary tract infections (UTIs), sepsis. Increased hospitalizations and treatment failures. Higher mortality in severe infections.	Continues to be a leading cause of community- and healthcare-associated infections globally. Outbreaks of carbapenemase-producing *E. coli* reported in healthcare settings worldwide.	[2],[3],[33],[35]
*K. pneumoniae*	Elevated resistance to critical antibiotics, including carbapenems. Emergence of pan-drug resistant strains. Widespread prevalence of carbapenemases (KPC, NDM, OXA-48).	Severe, often untreatable infections (pneumonia, bloodstream infections). Increased use of last-resort drugs, leading to further resistance development. High mortality rates, especially in immunocompromised patients.	Major driver of healthcare-associated outbreaks globally, particularly in ICUs. Reports of CRKP outbreaks in Europe and Asia.	[2],[3],[33],[35]
*S. aureus*	35% median resistance for MRSA. Increasing VISA and heterogeneous VISA (hVISA) strains. Linezolid and daptomycin resistance emerging.	Difficult-to-treat skin and soft tissue infections, pneumonia, bloodstream infections, endocarditis. Prolonged hospital stays and higher healthcare costs. Significant morbidity and mortality.	MRSA remains a predominant cause of hospital-onset infections in the U.S., with increases during the COVID-19 pandemic. Community-associated MRSA outbreaks continue.	[2],[3],[35]
*P. aeruginosa*	High intrinsic resistance. Increasing resistance to carbapenems, colistin, and novel β-lactam/β-lactamase inhibitor combinations. MDR and XDR strains prevalent.	Severe infections in immunocompromised patients (pneumonia, UTIs, surgical site infections). Limited treatment options for XDR strains. High mortality rates.	Frequent cause of ventilator-associated pneumonia and infections in cystic fibrosis patients. Outbreaks of MDR *P. aeruginosa* reported in healthcare facilities.	[3],[33],[35]
*A. baumannii*	Predominantly MDR and XDR, often resistant to nearly all available antibiotics. High rates of carbapenem resistance (OXA-23, OXA-40, OXA-58). Colistin resistance emerging.	Critically ill patient infections (pneumonia, bloodstream). Few to no effective treatment options for XDR strains. High mortality in critically ill patients.	A major threat in ICUs globally, responsible for severe outbreaks due to its persistence in the environment. Outbreaks of carbapenem-resistant *A. baumannii* common in war zones and healthcare settings.	[3],[33],[35]

CRKP—Carbapenem-resistant *K. pneumoniae*; ICU—Intensive care unit; MRSA—Methicillin resistant *S. aureus*; VISA—Vancomycin-intermediate *S. aureus*; hVISA—Heterogeneous VISA; COVID-19—Coronavirus disease of 2019; UTIs—Urinary tract infections; XDR—Extensively drug-resistant; MDR—Multidrug resistance.

### 4.1. Phenotypic resistance in major pathogens

The phenotypic expression of AMR, which manifests as the ability of a microorganism to grow in the presence of an antimicrobial agent, is a critical determinant of clinical failure. This resistance is not uniform across bacterial species but exhibits distinct patterns in key pathogens that frequently cause severe infections [89],[90]. The discovered genes responsible for this resistance are listed in Table 3.

**Table 3.** Recent (2020–2025) clinically relevant resistance genes.

Resistance Gene	Mechanism	Associated Pathogens	Notable Features/Examples	Reference
*bla*_NDM-5_	Carbapenemase (β-lactamase hydrolyzing carbapenems)	*E. coli*, *K. pneumoniae*	Confers high-level resistance to carbapenems; rapid global spread via IncX3 plasmids	[12],[32]
*bla*_OXA-181_	Carbapenemase (Class D β-lactamase)	*K. pneumoniae*, *E. coli*	Plasmid-mediated; associated with outbreaks in South Asia, Middle East, and Africa	[12],[32]
*mcr-9*	Phosphoethanolamine transferase (colistin resistance)	*Enterobacter cloacae*, *Salmonella* spp.	Mobile colistin resistance gene; detected in clinical and environmental isolates globally	[12],[32]
*tet*(X4)	Flavin-dependent monooxygenase (tigecycline resistance)	*E. coli*, *Acinetobacter* spp.	Inactivates tigecycline and eravacycline; plasmid-borne; first identified in China, now global	[12],[32]
aac(6′)-Ib-cr	Aminoglycoside acetyltransferase (modifies fluoroquinolones and aminoglycosides)	*E. coli*, *K. pneumoniae*	Plasmid-mediated; confers resistance to both aminoglycosides and ciprofloxacin	[32],[91]
*bla*_CTX-M-65_	ESBL	*E. coli*, *K. pneumoniae*	Confers resistance to third-generation cephalosporins; associated with foodborne and clinical outbreaks	[12],[32]
sul3	Dihydropteroate synthase variant (sulfonamide resistance)	*E. coli*, *Salmonella* spp.	Plasmid and transposon-borne; increasingly detected in animal and human isolates	[12]
*rpoB* S450L	RNA polymerase β-subunit mutation (rifampicin resistance)	*Mycobacterium tuberculosis*	High-level rifampicin resistance; identified via WGS in clinical isolates	[32],[91]
*gyrA* S83L/D87N	DNA *gyra*se mutations (fluoroquinolone resistance)	*E. coli*, *Salmonella* spp.	Common in MDR Enterobacterales; often co-occurs with plasmid-mediated resistance	[32],[91]
*walK*/walR	Two-component system mutations (vancomycin-intermediate resistance)	*S. aureus*	Mutations reduce vancomycin susceptibility; identified in clinical VISA isolates	[91]
*lon*	ATP-dependent protease mutation (eravacycline resistance)	*K. pneumoniae*	Mutations or Tn insertions upstream of *lon* gene linked to rapid eravacycline resistance	[12]
cfr(B)	23S rRNA methyltransferase (linezolid resistance)	*E. faecium*, *Staphylococcus* spp.	Plasmid-mediated; confers resistance to oxazolidinones, phenicols, lincosamides, pleuromutilins, streptogramin A	[32]
*bla_NDM-5_*	Carbapenemase (β-lactamase hydrolyzing carbapenems)	*E. coli*, *K. pneumoniae*	Confers high-level resistance to carbapenems; rapid global spread via IncX3 plasmids	[12],[32]

ESBLs—Extended spectrum beta lactamases; MDR—Multidrug resistance; VISA—Vancomycin-intermediate *S. aureus*; rRNA—Ribosomal ribonucleic acid.

Biofilm-enhanced resistance complicates the management of urinary tract infections caused by *E. coli*, which exhibits escalating β-lactam resistance linked to *bla*_CTX-M-15_ and *bla*_NDM-5_ carbapenemase genes [90]. Recent outbreaks include MDR uropathogenic *E. coli* (UPEC) in Somalia, showing >90% ceftriaxone resistance driven by ESBL production and efflux pump overexpression [91],[92]. UPEC isolates from European countries show variable resistance to ceftriaxone and other cephalosporins, with resistance levels to third-generation cephalosporins ranging from 5.3% (Germany) to 37.6% (France).

In England, resistance to third-generation cephalosporins in hospitalized patients ranges from 13.8% to 21.3%. UPEC susceptibility to last-resort antibiotics remains high; however, increasing resistance to commonly used antibiotics, such as ampicillin and tetracycline, has been documented in Europe and North America, highlighting the need for continuous vigilance [92]. Emerging molecular epidemiology studies indicate that prevalent MDR UPEC lineages, such as ST131, are associated with resistance to ceftriaxone and other β-lactams [93].

Rising rifampicin resistance (projected 46.7% by 2027) and emerging vancomycin tolerance (16.7% in 2022) are demonstrated by *S. aureus*, with mecA-mediated methicillin resistance remaining prevalent [93],[94]. Rifampicin resistance mediated by rpoB mutations in *S. aureus* is emerging globally, promoting reduced susceptibility not only to rifampicin but also to cross-resistance to vancomycin and daptomycin, thus threatening last-line therapies worldwide [94].

Vancomycin intermediate *S. aureus* (VISA) and vancomycin-resistant *S. aureus* (VRSA) have been reported across regions, including Asia and North America, with varying reduced susceptibility rates, showing an increasing trend [95]. The prevalence of methicillin-resistant *S. aureus* (MRSA) remains high, with *the mecA* gene detected in over 60% of *S. aureus* isolates in several studies globally, including in Europe and North America, confirming that mecA-mediated resistance is a global issue [96].

Outbreaks in Nigeria (2022) revealed that *K. pneumoniae* has universal resistance to ceftriaxone and colistin, with carbapenemase genes (*bla*_KPC_ and *bla*_OXA-48_) detected in 74% of the isolates [91]. In Asia, hypervirulent *K. pneumoniae* strains predominantly remain susceptible to antibiotics, but MDR strains carrying carbapenemase genes (such as *bla*_KPC_, *bla_NDM_*, and *bla*_OXA-48_) are increasingly reported, particularly in China and Taiwan, impacting clinical outcomes [97].

European data show a growing prevalence of carbapenem-resistant *K. pneumoniae* (CRKP), with some countries reaching 40–60% resistance rates in recent years. ESBL production and multidrug resistance are common in Mediterranean and Eastern Europe [98]. In North America, resistance phenotypes of *K. pneumoniae* in urinary tract infection isolates demonstrate increasing resistance to nitrofurantoin, trimethoprim/sulfamethoxazole, fluoroquinolones, and extended-spectrum β-lactamase production, with multidrug resistance trends increasing over time [99]. Carbapenemase genes, such as *bla_KPC_* and *bla_OXA-48_*, are prevalent in clinical isolates from these regions, highlighting the global dissemination of resistance elements [97],[98].

Alarming resistance in Bangladesh (2025) has been observed, with *P. aeruginosa* demonstrating 98.1% aztreonam resistance and 74.1% carbapenem resistance, linked to mexAB-oprM efflux overexpression and *ampC* mutations [95]. In Europe, surveillance data from 2019 to 2023 show high antimicrobial resistance in *P. aeruginosa* bloodstream and invasive infections, with notable resistance to carbapenems and other drug classes, although recent years have seen a decline in certain resistance patterns. Carbapenem resistance remains a significant concern, reported at higher rates than *Klebsiella pneumoniae* in some settings [100].

A systematic review noted an overall *P. aeruginosa* resistance to meropenem of approximately 28.6 %, with rates varying by country in Europe and North America. Ciprofloxacin resistance was approximately 46.5%, with trimethoprim-sulfamethoxazole showing up to 75% resistance in some studies [101]. Resistance to key antibiotics in *P. aeruginosa* influences empirical treatment success and prompts the need for susceptibility testing before making therapeutic decisions [102].

### 4.2. Impact on treatment outcomes

Therapeutic failures due to AMR caused 4.71 million global deaths in 2021, with MDR infections increasing mortality by 2–3 fold in patients with sepsis [103]. Carbapenem-resistant *K. pneumoniae* outbreaks in Somalia resulted in 57% mortality among neonates, whereas MDR *P. aeruginosa* pneumonia cases required 28-day extended ICU stays [104],[105]. Economically, AMR costs sub-Saharan Africa $65 billion annually owing to prolonged hospitalization and second-line therapies [103],[106]. In Banadir, Somalia, pediatric MDR bloodstream infections increased healthcare costs by 300% compared to susceptible strains [107].

### 4.3. Diagnostic challenges

An accurate and timely diagnosis of AMR is critical for guiding appropriate therapy and implementing effective infection control measures. However, diagnostic methodologies have notable limitations, prompting the need for more advanced solutions. Traditional phenotypic antimicrobial susceptibility testing (AST), considered the gold standard, often relies on bacterial culture, which can be time-consuming, typically taking 24–72 hours or more for definitive results [108],[109].

The delay in culture results often necessitates the use of broad-spectrum empirical antibiotics, which can contribute to further development of resistance if the causative pathogen is susceptible to a narrower-spectrum agent [110],[111]. Furthermore, slow-growing or fastidious organisms pose challenges to standard culture-based AST [103],[112],[113]. Phenotypic tests may also struggle to detect all resistance mechanisms, particularly those involving complex genetic interactions and novel resistance genes [113].

Conventional susceptibility testing fails to detect heteroresistance in *S. aureus* biofilms and carbapenemase-negative Enterobacterales with porin mutations [114]. Automated systems misinterpret *Pseudomonas* spp. susceptibility to piperacillin-tazobactam because of the inoculum effect [105]. Advances have improved the detection and characterization of drug-resistant pathogens in the environment. These include multiplex PCR for the rapid detection of *K. pneumoniae* virulence genes (*mrkD* and *K2*) and resistance markers (*bla*_NDM-1_) [104]. The identification of *E. coli* ST131 clonal expansion in outbreak settings has been achieved using WGS [115], and MALDI-TOF MS coupled with machine learning can predict *P. aeruginosa* resistance profiles within 4 h [105].

Molecular methods can rapidly detect specific resistance genes or mutations (within hours) and can often be applied directly to clinical specimens without culture. However, they identify only known resistance markers and fail to detect novel or complex resistance mechanisms, such as non-enzymatic pathways or gene expression levels, potentially leading to overcalling resistance [26],[32]. However, they do not provide minimum inhibitory concentrations (MICs), limiting the ability to tailor precise susceptibility profiles, and cannot fully replace phenotypic methods in clinical decision-making [26]. Although molecular panels targeting multiple pathogens and resistance genes exist, their sensitivity can be limited, and they may miss resistance genes outside the tested panel, thereby underestimating resistance [32].

Advanced molecular diagnostics, such as WGS, remain costly relative to traditional phenotypic tests, with per-test costs reported to be between approximately $70 and $470. This cost is largely due to expenses related to sequencing, data storage, bioinformatics analysis, and the need for specialized equipment and expertise [116]. The financial burden and infrastructural demands restrict the implementation of WGS primarily to high- and upper-middle-income countries with reliable supply chains and laboratory capacity. Analytical pipelines for WGS require skilled bioinformatics personnel and computational resources, which may be unavailable or insufficiently developed in many clinical settings, particularly in resource-limited environments [117].

Accessibility in low-resource settings is challenged by the initial setup cost, maintenance of sequencing platforms, supply chain limitations for reagents, and a lack of trained personnel [118]. Simpler molecular methods, such as PCR, remain more accessible because of their relatively lower cost and technical demand; however, they offer limited scope. Conversely, WGS offers comprehensive data but is less feasible in these settings without targeted investments and capacity building [117],[118]. The technological complexity and need for continuous updates in molecular diagnostic assays to keep pace with emerging resistance genes can be difficult to maintain in low-resource laboratories [117].

In resource-limited settings, cost-effective diagnostic approaches can help overcome the barriers posed by expensive technologies such as WGS. Low-cost genotyping tools, such as agarose-MAMA, enable SNP-based pathogen typing using standard laboratory equipment in developing countries [119]. Programs such as SeqAfrica have demonstrated decentralized AMR surveillance through regional sequencing centers across Africa, thereby supporting local data generation [120],[121]. Portable sequencing platforms, such as nanopore technology, facilitate genomic epidemiology in resource-poor environments [122],[123].

## 5. Interplay of resistance mechanisms: Synergy and trade-offs

Often, AMR mechanisms coexist and interact in bacterial populations, resulting in complex synergistic effects that boost their survival under various selective pressures. Co-resistance, in which multiple resistance genes are physically linked to MGEs, such as plasmids or integrons, facilitates the simultaneous horizontal transfer of traits conferring resistance to antibiotics and other agents such as heavy metals [124],[125].

For example, *Salmonella enterica* serotype Typhi harbors conjugative plasmids (pHCM1) encoding both mercury resistance genes (*merR*) and antibiotic resistance genes for ampicillin, chloramphenicol, and sulfonamides, enabling the co-selection and rapid dissemination of MDR. Cross-resistance occurs when a single mechanism, such as multidrug efflux pumps (AcrAB-TolC in *E. coli*), expels structurally unrelated antibiotics and toxic metals, broadening the resistance spectrum [126],[127]. Furthermore, the co-regulation of resistance gene expression and biofilm formation enhances bacterial resilience by promoting HGT within biofilms and coordinating stress responses [126].

However, the acquisition of resistance often imposes fitness costs, such as reduced growth rates or virulence. Compensatory evolution through secondary mutations can mitigate these costs without sacrificing resistance levels, as observed for plasmid-borne resistance in *E. coli* and chromosomal mutations in *P. aeruginosa* [128]. Notably, resistance to bacteriophages can entail trade-offs, including diminished virulence and re-sensitization to antibiotics, a phenomenon exploited in phage-steering strategies to reverse resistance and attenuate pathogenicity [129].

## 6. Integrating advanced molecular diagnostics and one health approaches to combat AMR

The escalating complexity of AMR, driven by genetic evolution and HGT across ecological niches, necessitates the integration of advanced molecular diagnostics within the One Health Framework. Cutting-edge techniques, such as WGS, metagenomics, and rapid PCR-based assays, enable the precise real-time identification of resistance determinants, their genetic contexts, and transmission dynamics [32],[130]. These tools facilitate the early detection of emerging resistance genes, track clonal outbreaks, and inform tailored antimicrobial stewardship interventions, thereby improving clinical outcomes and curbing the spread of resistance [65],[131].

Addressing the complex factors contributing to AMR requires a One Health strategy that acknowledges the interdependence of human, animal, and environmental health. Australia's Antimicrobial Use and Resistance in Australia (AURA) program collects antimicrobial use and resistance data from hospitals, aged care facilities, laboratories, and communities across the country. The Australian Passive AMR Surveillance (APAS) program has captured over 124 million susceptibility results, providing insights into resistance trends and supporting AMR stewardship efforts. CARAlert monitors high-priority organisms threatening last-line antibiotics through voluntary data submission, enabling rapid outbreak detection. The HOTspots pilot program operates in northern Australia, providing real-time AMR data to clinicians for effective treatment in remote areas [132].

The U.S. The National Antimicrobial Resistance Monitoring System (NARMS), established in the mid-1990s, exemplifies early cross-sector collaboration for monitoring AMR in foodborne and enteric bacteria and involves the CDC, FDA, and USDA. It tracks resistance trends linked to the use of food animals, providing insights into transmission between animals, the environment, and humans [133]. The WHO's Global Antimicrobial Resistance & Use Surveillance System (GLASS) offers a global platform for integrating multi-sector data, emphasizing a One Health approach with systems such as ESBL-E, which tracks resistance in humans, animals, and the environment [10].

The U.S. CDC and FDA's collaboration in the 1990s led to the early development of integrated AMR surveillance in the food production chain, laying the groundwork for broader One Health initiatives, such as the European Centre for Disease Prevention and Control (ECDC) surveillance programs that incorporate animal and environmental sectors [133],[134]. African One Health AMR studies focus on regional data synthesis, emphasizing the interconnectedness of resistance in humans, livestock, and environmental reservoirs, particularly in resource-limited settings [135].

France's surveillance programs for antibiotic use and resistance, integrated across human and veterinary sectors, exemplify nationwide efforts to map and control AMR from a One Health perspective [136]. The Nigerian National AMR Action Plan integrates surveillance, stewardship, and infection prevention strategies across the human and animal health sectors, aligning with the WHO guidelines for a multisectoral approach [137].

Resistance genes and MGEs circulate freely among humans, livestock, wildlife, and environmental reservoirs, such as water and soil, thereby amplifying this risk [138]–[140]. Surveillance programs incorporating molecular diagnostics across these sectors have revealed shared resistance mechanisms, including plasmid-mediated colistin resistance (*mcr* genes) and ESBLs, highlighting the need for coordinated, cross-sectoral strategies [141],[142].

By harmonizing molecular surveillance data with epidemiological and ecological insights, the One Health initiative can guide policy development, optimize infection prevention and control, and promote responsible global antimicrobial use [143],[144]. Ultimately, leveraging advanced diagnostics within the One Health paradigm is indispensable for the effective containment of AMR and safeguarding the efficacy of antimicrobials in future generations of humans and animals.

These comprehensive global surveillance studies demonstrate the value of coordinated data collection, sharing, and response strategies that span several sectors. They facilitate early detection, track transmission pathways, and inform targeted interventions to curb the spread of resistance genes, such as *mcr* family plasmids and ESBLs, exemplifying a robust One Health paradigm [1],[133],[137].

## 7. Future directions and strategies to combat AMR

Effective AMR containment requires integrated surveillance and stewardship programs to monitor resistance trends and optimize the use of antibiotics. Global initiatives emphasize genomic surveillance to detect emerging resistance genes and mobile elements, thereby facilitating targeted interventions [126],[145]. Novel therapeutic approaches are gaining momentum, including inhibitors targeting resistance pathways (e.g., SOS response inhibitors), immuno-antibiotics that disrupt bacterial metabolic networks, and agents that neutralize biofilms to enhance antibiotic penetration [145].

Bacteriophage therapy represents a promising alternative, particularly against MDR pathogens such as *E. coli*, *P. aeruginosa*, *A. baumannii*, and *S. aureus*. Studies have demonstrated the potent lytic activity and biofilm disruption capabilities of phages, with in vivo models showing improved survival and reduced bacterial load compared to antibiotics alone [125]. Combination therapies leveraging phage-antibiotic synergy exploit fitness trade-offs to resensitize bacteria and reduce their virulence [129].

Prospects for reversing resistance include “phage steering” to select less virulent, antibiotic-susceptible bacterial phenotypes and the development of molecules targeting compensatory mechanisms that sustain resistance without fitness penalties [128],[129]. Continued research integrating molecular insights into clinical strategies is essential to overcome the evolving threat of AMR.

## 8. Conclusion

A critical and escalating global health threat is posed by AMR, which is driven by complex mechanisms including genetic mutations, horizontal gene transfer, and bacterial adaptive strategies such as biofilm formation and efflux pump overexpression. These dynamics accelerate the emergence and spread of resistant strains, compounded by the influence of genetic backgrounds, mobile genetic elements, and environmental factors. Addressing AMR requires immediate and actionable strategies, such as robust surveillance under One Health frameworks, antimicrobial stewardship to reduce inappropriate antibiotic use, and improved rapid diagnostics to guide targeted therapy and minimize the broad-spectrum misuse of antibiotics. Long-term solutions focus on innovative therapies, including phage therapy, biofilm-disrupting agents, pathway inhibitors, and vaccine development, as well as sustained global collaboration and investment. Current data reveal alarming impacts: One in six bacterial infections globally exhibits resistance, contributing to over 1.27 million deaths annually, with projections of up to 10 million deaths by 2050 if not controlled. Thus, only through an integrated, dual approach of immediate control measures and breakthrough innovations, supported by coordinated multisectoral efforts, can the spread of resistance be curtailed and antimicrobial efficacy preserved for future generations. Future research must prioritize the development of novel inhibitors that target key resistance mechanisms and pursue innovative therapies, such as phage therapy, biofilm-disrupting agents, and vaccines, all supported by sustained global collaboration to curtail the proliferation of resistant strains and preserve future antibiotic efficacy.

## Use of AI tools declaration

The authors declare they have not used Artificial Intelligence (AI) tools in the creation of this article.
